# The association of sociodemographic characteristics with work disability trajectories during and following long-term psychotherapy: a longitudinal register study

**DOI:** 10.1007/s00127-023-02523-y

**Published:** 2023-07-11

**Authors:** Sanna Selinheimo, Kia Gluschkoff, Johanna Kausto, Jarno Turunen, Aki Koskinen, Ari Väänänen

**Affiliations:** 1 Finnish Institute of Occupational Health, Työterveyslaitos, PO Box 18, 00032 Helsinki, Finland; 2 Department of Psychology and Logopedics, University of Helsinki, Helsinki, Finland

**Keywords:** Mental health, Health inequity, Psychotherapy outcome, Work disability, Trajectories

## Abstract

**Purpose:**

This register-based study examined the trajectories of depression or anxiety disorder-related work disability during and following long-term psychotherapy and identified sociodemographic factors that indicate membership in different trajectory groups.

**Methods:**

Data were drawn from national registers (Statistics Finland, Social Insurance Institution of Finland). Participants included a random sample of Finnish working-age individuals (18–55 years) who started psychotherapy treatment between 2011 and 2014 and were followed for 5 years: 1 year before and 4 years after the onset of psychotherapy (*N* = 3 605 individuals; 18 025 person-observations across five time points). Group-based trajectory modeling was applied to assign individuals to work disability trajectories by the number of annual mental health-related work disability months. Multinomial logistic regression was used to examine the associations between trajectory group membership and baseline sociodemographic factors of age, gender, occupational status, and geographical area of residence.

**Results:**

Four mental health-related work disability trajectories were identified: stable very low (72%), decrease (11%), persistent low (9%) and persistent high (7%). Those with older age, female gender, lower occupational status, and living in sparsely populated geographical areas were more likely to belong to the most unfavorable trajectory group of persistent high work disability. The presence of multiple risk characteristics substantially increased the probability of belonging to the most adverse trajectory group.

**Conclusions:**

Sociodemographic factors were associated with the course of mental health-related work disability in association with psychotherapy. Rehabilitative psychotherapy does not function as an equal support resource for work ability in all parts of the population.

**Supplementary Information:**

The online version contains supplementary material available at 10.1007/s00127-023-02523-y.

## Introduction

Depression and anxiety disorders are among the leading causes of work disability globally [1–3]. These disorders have a significant impact on the ability to study or work, the number of sick leaves, direct and indirect health expenditures, work productivity, and societal burden [4–8]. Occupational classes have been shown to differ in the risk of depression and anxiety-related work disability [4, 9, 10] and such differences in the risk increase over the life course [11, 12].

Psychotherapeutic treatments are widely acknowledged as effective for depression and anxiety disorders symptom reduction [13]. The treatments remove obstacles to workability and reduce mental health-related sickness absences and work disability [14, 15]. However, these person-centered treatments may not be equally efficient in improving functioning between occupations with varying work-related risk factors. Adverse work characteristics have been associated with common mental health disorders: psychosocial stressors such as imbalanced job design (i.e., low job control and high demands), occupational uncertainty and perceived lack of value and respect in the workplace (i.e., effort-reward imbalance) are linked with higher mental distress [16, 17] and the risk for work disability due to poor work characteristics have proved to be pronounced among low occupational status individuals [18, 19]. Thus, as individuals with lower occupational status are at greater risk of being exposed to such adverse work characteristics [20] psychotherapeutic treatments might not be as effective for them in comparison to individuals with higher occupational status. However, to date, information is scarce on occupational status’ role in response to psychotherapy treatment when aiming to improve and maintain the workability of those at high risk of work disability because of mental disorders.

Furthermore, previous research has shown significant regional variation in mental health-related work disability [21]. Studies have observed differences in mental health care practices between urban and rural areas suggesting more detrimental effects for those living outside urban areas: together with different clinical practices [21, 22] it might be that in a country like Finland, long regional distances might lead to suboptimal treatment use. Furthermore, the influence of mental distress on work disability might be more pronounced in more disadvantaged areas with fewer occupational opportunities. Thus, previous studies suggest an existing association between a geographical area of residence and mental health outcomes but whether outcomes differ among those who receive psychotherapy treatment remains unknown.

Studies on the effectiveness of psychotherapeutic treatments have mainly focused on examining changes in outcomes of the mean level of change [23–25]. Such methodological approaches may, however, overlook subgroups of clients who are unable to benefit from psychotherapy or whose pattern of change during the treatment differs from the mean [23, 24]. Previous studies have shown that clinical characteristics such as baseline symptom severity and psychological functioning influence clients’ trajectories of change so that some improve at the initial phases of the treatment whereas others progress more gradually or even show a decrease in functioning during the treatment [24, 26, 27]. The advantages and resources related to occupational status or geographical area might influence this variation between treatment trajectories. However, while the interest in trajectories of change has increased, there is a lack of information on such contextual factors intervening change process.

Person-centered approaches have been designed to address this variability across clients’ responses to the treatment and to reveal patterns of change in a longitudinal setting [28, 29]. To assess whether occupational status or geographical area influences clients’ ability to benefit from the treatment, such an analytic approach could be used to determine a continuous change process over time among groups with different statuses. Improving understanding of how psychotherapy works among different groups of clients may help to improve the practices when aiming to intervene in socioeconomic inequalities in mental health-related work disability.

In Finland, rehabilitative psychotherapy is the major single form of publicly provided rehabilitation. It is targeted at those at risk for disability to work or study because of mental disorders [30]. It is granted for a yearly period (maximum 3 years), a maximum of 80 sessions per year and 200 sessions per 3 years and could be cognitive, cognitive behavioral, cognitive analytic, psychodynamic, integrative, solution-focused or family therapy. From 2011 on it has been statutorily granted for all at risk. Despite the statutory status, a patient needs a psychiatrist’s referral to psychotherapy. The referral must be preceded by a minimum of three-month follow-up to assess whether first-line treatments (mainly pharmacological or in some cases short psychological counseling) are efficient for improving functioning and workability. After the referral, a patient needs to find an available psychotherapist from the private healthcare sector. Results from two recent studies suggest that rehabilitation was associated with somewhat better labor market outcomes [31, 32]. However, there is no information on whether occupational status or area of residence influence work disability progress during and after rehabilitation and to what extent disability trajectories have differed among individuals who started rehabilitative psychotherapy after it became accessible to all eligible for the benefit. Our study extends research on this issue by identifying work disability trajectories over 1 year before and 4 years after the onset of rehabilitative psychotherapy using nationally representative data. Further, to assess whether there are social position-related inequalities in achieving improvement during therapy examined the associations of occupational status and area of residence with differences in work disability trajectories.

### Aims of the study and research questions

Our aim was to (i) ascertain psychotherapy clients’ typical depression or anxiety-related work disability trajectories and (ii) examine whether sociodemographic factors (age, gender, occupational status, and geographical area of residence) are associated with work disability trajectory group membership. Our research questions were: “What kind of work disability trajectories can be recognized among a cohort of rehabilitative psychotherapy users?” and “Do sociodemographic factors predict the users’ trajectory group membership?”.

### Hypothesis

Based on earlier studies [18–22], we hypothesized that low occupational status and living in sparsely populated areas together with older age are associated with poor and prolonged work disability outcomes during and following the treatment.

## Methods

### Sample

We used data from the Rise of Mental Vulnerability project [32, 33], in which cohorts of 33% random samples of the working-age population in the census of 2010, *N* = 772 663 were drawn from the Statistics Finland population database. We selected 18–55-year-old individuals who began rehabilitative psychotherapy between 2011 and 2014 (*N* = 10,497). We excluded those who had had discretionary rehabilitative psychotherapy before the legislation change in 2011 but whose rehabilitation was still ongoing (*n* = 2240), those not in paid employment (students, unemployed, self-employed persons) during the study period (from the year before the onset of psychotherapy until a three-year follow-up, *N* = 4641) as well those who were on permanent disability pension before the onset of psychotherapy (*N* = 11), resulting in a sample size of 3605 individuals.

## Measures

### Work disability

The primary outcome was work disability due to depression and anxiety disorders (ICD-10 codes F32-33 and F40-F43), measured at five time points relative to psychotherapy onset: the year preceding onset (baseline), the year of onset, and one through 3 years following the onset of psychotherapy. For each individual, the annual number of disability months (0–12) was calculated based on their totaled annual compensated sickness absence (SA) and disability pension (DP) days in these diagnostic categories. First, the annual number of compensated SA days due to depression or anxiety disorders was extracted from the registers of the Social Insurance Institution. Shorter SA periods (lasting less than ten working days) are not compensated by the Social Insurance Institution and therefore data on shorter absences were not available for this study. Second, the annual number of compensated DP days due to depression or anxiety disorders was extracted from the registers of the Finnish Centre for Pensions. Part-time DP days were converted into whole days so that two part-time DP days counted as one whole day. The total number of work disability days was converted into months: no work disability months for ≤ 7 annual work disability days; 1 month for 8–29 days; 2 months for 30–59 days and so forth (see [9]).

#### Rehabilitative psychotherapy

The onset of psychotherapy treatment was determined as the year (between 2011 and 2014) when the individual first received reimbursement for psychotherapy from the Social Insurance Institution of Finland. The duration of psychotherapy was calculated by using the information on reimbursements for psychotherapy during the years following psychotherapy onset. Because very few participants (3%) had a psychotherapy duration of more than four calendar years, the variable was top-coded at 4 years.

#### Sociodemographic characteristics

Data on sociodemographic characteristics were obtained from the year preceding the onset of psychotherapy (baseline) from the Statistics of Finland register. These included age, gender, occupational status, and geographical area of residence. Occupational status was coded according to the socioeconomic group into upper-level and lower-level employees and manual workers. Regarding the geographical area of residence, the Finnish health care system is divided into five regional specialized university hospital districts with 700 000–2 200 000 inhabitants. Geographical area was classified according to the university hospital districts, located in southern (Helsinki University Hospital), central (Tampere University Hospital), western (Turku University Hospital), eastern (Kuopio University Hospital) and northern (Oulu University Hospital) Finland.

### Statistical analysis

We conducted group-based trajectory analysis using the STATA plugin TRAJ [34] to identify subgroups of individuals who follow similar courses of work disability due to mental disorders before, during and after rehabilitative psychotherapy. For the analysis, time was normalized so that time t0 corresponds to the onset of psychotherapy. The trajectories of the annual number of work disability months from t-1 (baseline) to t3 were modeled with a zero-inflated Poisson distribution. In the zero-inflated Poisson model with the TRAJ plugin, both an “order” and an “iorder” option can be specified. Whereas the order option refers to trajectory shapes, the iorder option refers to the degree to which zero-inflation in the trajectory groups varies over time (i.e., the pattern of excessive zeros). We initially fitted models with an intercept and a linear and quadratic term for two to five trajectory solutions. A combination of several criteria was used to determine the optimal number of groups: the Bayesian Information Criterion (BIC), trajectory group size (minimum group size of 5%), and whether the model captured new distinctive features of the data (i.e., whether increasing the group size resulted in new distinct trajectories) [35]. We chose parsimony over complexity in model selection to avoid performing a high number of group comparisons at the next stage of the analysis, where the associations between client characteristics and trajectory group membership were examined. Once the optimal number of groups was established, the shape of the trajectory for each group was refined to its final form by testing higher-order (cubic) terms for the trajectories and higher-order terms for the zero-inflation and removing the highest-order terms that were nonsignificant. After this, individuals were assigned to a trajectory group to which they most likely belonged based on estimates of their posterior probability of group membership. Although it would have been possible to estimate the probability of latent trajectory group membership and the effects of client characteristics on the group membership probability in a joint model, we chose to estimate the trajectory groups in their “pure” form. This two-step approach, where the associations between risk factors and trajectory group membership are examined after the groups are identified, is widely used in the literature. We performed an analysis of variance for continuous variables and *χ*^2^ tests for categorical variables to investigate whether the trajectory subgroups differed in their sociodemographic characteristics. Multinomial logistic regression was performed to estimate the multivariate associations between client characteristics and membership in various disability trajectories. Age, gender, occupational status, and geographical area of residence were entered as predictors of trajectory group membership. The regression models were adjusted for the duration of psychotherapy and the calendar year of the onset of psychotherapy. The resulting estimates were obtained using the most favorable trajectory as a reference category and expressed as relative risk ratios (RRR, sometimes called a multinomial odds ratio) and 95% confidence intervals (CIs). The interpretation of the RRR is that for a unit change in the predictor variable, the odds of the outcome relative to the reference group are expected to change by its respective RRR estimate, holding all other variables in the model constant. The analyses were conducted on STATA 17 and visualizations were produced using R 4.0.5 with ggplot2 [36].

## Results

A total of 3605 participants were included (79% female, mean age 37 years), contributing 18,025 person-year-observations across five time points from t−1 to t3. Of the participants, 24.9% had started psychotherapy in 2011 (*n* = 896); 24.2% in 2012 (*n* = 872); 24.9% in 2013 (*n* = 899); and 26.0% in 2014 (*N* = 938). The mean duration of psychotherapy was 3.23 years.

**Fig. 1:**
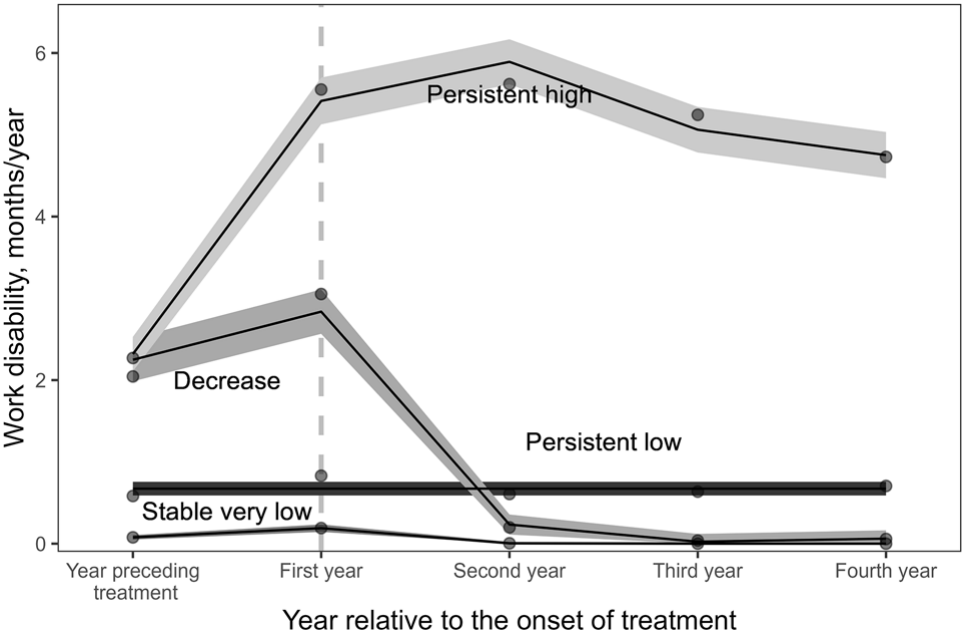
Depression or anxiety-related work disability trajectories were identified using group-based trajectory modeling. The solid lines are based on the parameter estimates of the model (with a 95% confidence interval as shaded area), whereas the dots are calculated with the actual data where the responses are weighted based on posterior probabilities of group membership. The dashed vertical line indicates the onset year of psychotherapy

Table 1 presents model statistics for the tested trajectory models (see Supplementary material for a more detailed description of the model selection and refinement process). We identified four distinct trajectory groups of mental health-related work. The first, most favorable trajectory group, “stable very low” (*n* = 2 604, 72.2% of participants), had a very low work disability trajectory across the follow-up. The second trajectory group, “persistent low” (*n* = 405, 11.2% of participants), had a low but constant work disability trajectory across the follow-up. The third group, “decrease” (*n* = 328, 9.1%), showed a persistent decline from a higher baseline level of work disability to a lower level of work disability starting in the second year of psychotherapy. The fourth group, “persistent high” (*n* = 268, 7.4%), had the highest level of work disability at the onset of psychotherapy. In this unfavorable work disability trajectory group, work disability increased at the onset of therapy and remained significantly higher than in other groups during the follow-up. See Fig. 1 for a visual representation of the trajectories and Table 2 for the characteristics of participants by trajectory groups. The groups differed on all sociodemographic factors except occupational status.

**Table 1:** Model statistics for the tested trajectory models

Order	Iorder	BIC (*N* = 3605)	Entropy	Group 1 (%)	Group 2 (%)	Group 3 (%)	Group 4 (%)	Group 5 (%)
22	0	− 13122.25	0.755	75.40	24.60			
222	0	− 12659.74	0.704	66.32	20.89	12.79		
2222	0	− 12448.25	0.721	68.93	13.79	9.46	7.82	
22,222	0	− 12424.82	0.597	61.05	13.40	10.35	8.52	6.69
0222	0	Did not converge						
0333	0	− 12439.75	0.718	69.18	14.04	8.57	8.21	
0333	3	− 12416.19	0.731	69.54	13.87	8.40	8.18	
0333	0003	− 12294.45	0.660	72.45	11.71	9.13	6.71	
Final model								
0233	0003	− 12296.29	0.697	72.23	11.23	9.10	7.43	

The order option refers to trajectory shapes and the iorder option refers to the degree to which zero-inflation in the trajectory groups varies over time (i.e., the pattern of excessive zeros). Entropy is a measure of classification quality. Entropy values range from 0 to 1, with higher values indicating the more accurate assignment of individuals into trajectory groups based on posterior probabilities of classification. The average posterior probabilities in the final model were 0.85 (group 1), 0.88 (group 2), 0.75 (group 3) and 0.94 (group 4). Whereas the order option refers to trajectory shapes, the iorder option refers to the degree to which zero-inflation in the trajectory groups varies over time (i.e., the pattern of excessive zeros)

*BIC* Bayesian information criterion

**Table 2:** Characteristics of participants at baseline (t−1, the year preceding the onset of psychotherapy) and by trajectory groups

	Total sample at baseline	Group 1 (stable very low)	Group 2 (persistent low)	Group 3 (decrease)	Group 4 (persistent high)	*P* value for differences*
*N* (%)	3605 (100%)	2604 (74.2%)	405 (11.2%)	328 (9.1%)	268 (7.4%)	
Age (mean (SD))	37.24 (9.32)	36.11 (9.09)	38.30 (9.24)	39.92 (9.00)	43.44 (8.67)	< 0.001
Gender						< 0.001
Male	772 (21.4%)	599 (23.0%)	65 (16.1%)	66 (20.1%)	42 (15.7%)	
Female	2833 (78.6%)	2005 (77.0%)	340 (84.0%)	262 (79.9%)	226 (84.3%)	
Occupational status						0.097
Upper-level employee	1472 (40.9%)	1103 (42.4%)	141 (34.8%)	125 (38.1%)	105 (39.2%)	
Lower-level employee	1813 (50.3%)	1272 (48.9%)	224 (55.3%)	176 (53.7%)	141 (52.6%)	
Manual worker	318 (8.8%)	229 (8.8%)	40 (9.9%)	27 (8.2%)	22 (8.2%)	
Geographical area						< 0.001
Southern	1714 (47.6%)	1317 (50.6%)	167 (41.3%)	146 (44.5%)	84 (31.3%)	
Western	484 (13.4%)	350 (13.4%)	45 (11.1%)	50 (15.2%)	39 (14.6%)	
Central	540 (15.0%)	358 (13.8%)	78 (19.3%)	59 (18.0%)	45 (16.8%)	
Eastern	476 (13.2%)	316 (12.1%)	69 (17.1%)	36 (11.0%)	55 (20.5%)	
Northern	390 (10.8%)	263 (10.1%)	45 (11.1%)	37 (11.3%)	45 (16.8%)	
Duration of psychotherapy (mean (SD))	3.23 (0.92)	3.22 (0.92)	3.28 (0.93)	3.07 (0.92)	3.39 (0.90)	0.278

*Southern* Helsinki University Hospital district, *Western* Turku University Hospital district, *Central* Tampere University Hospital district, *Eastern* Kuopio University Hospital district, *Northern* Oulu University Hospital district, *SD* standard deviation

*Analysis of variance for continuous variables and χ^2^ tests for categorical variables

**Fig. 2:**
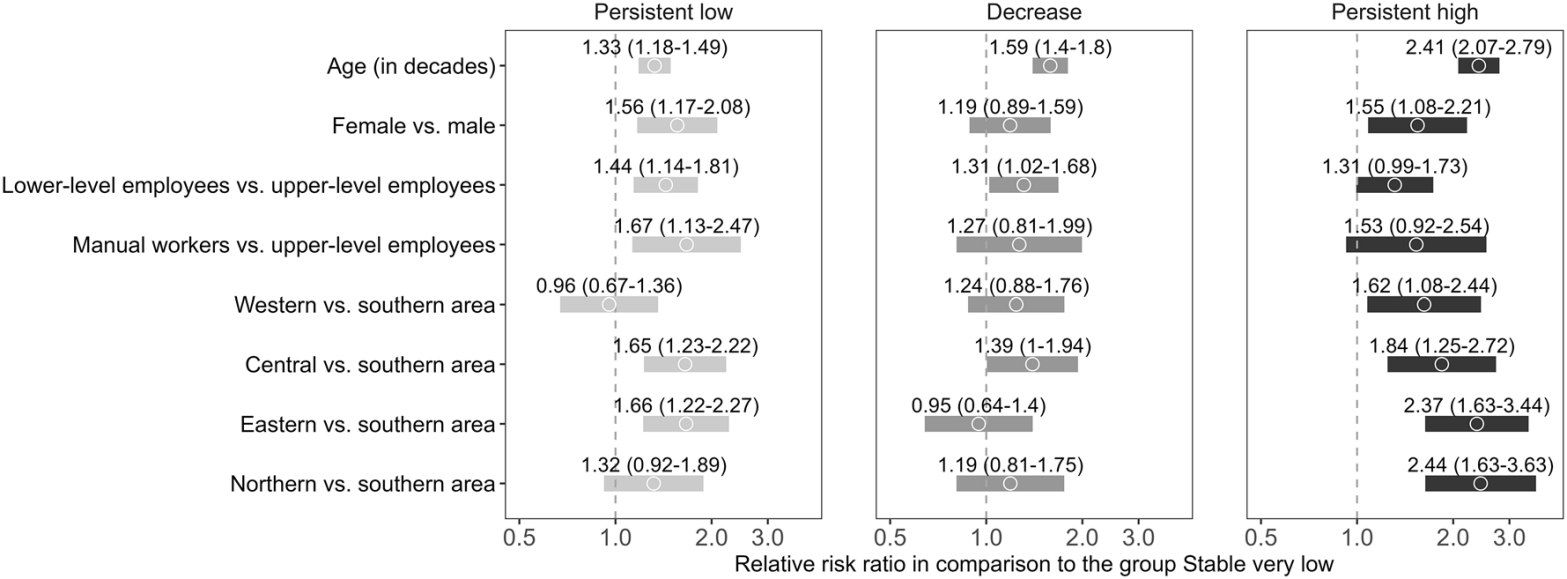
The associations between psychotherapy client background characteristics and membership in “Persistent decrease”, “Persistent low” and “Persistent high” work disability trajectories with membership in “Stable very low” as the reference category. Dots indicate relative risk ratios (i.e., multinomial odds ratios) and the bars indicate 95% confidence intervals

Overall, with regards to our hypothesis, the results of multinomial logistic regression (Fig. 2) showed that low occupational status and living in sparsely populated areas and older age are associated with poor work disability outcomes. In addition to our hypothesis, female gender was associated with poor work disability outcomes: relative to the stable very low group, higher age, female gender, manual or lower-level occupational status, and Central area of residence were associated with membership in the persistent low trajectory group. Furthermore, as for the comparison between stable very low and the persistent high trajectory group, higher age, female gender, manual or lower-level occupational status, and residence outside the Southern area, especially in the Northern or Eastern area of residence were associated with an increased risk of membership in the persistent high group. As for the comparison between stable very low and persistent low, again higher age, lower lever occupational status, and residence outside the Southern area, especially in the Eastern area or Central geographical area of residence were associated with an increased risk of membership in the persistent high group. Based on non-overlapping confidence intervals, particularly older age differentiated the persistent high from the decrease or stable very low groups and Easter and Northern area of residence from the decrease group (see Fig. 2). There were also differences in the duration of psychotherapy between the groups. Compared with the stable very low trajectory group, the treatment duration was shorter in the decrease (RRR = 0.86, 95% CI 0.76–0.97) and longer in the persistent high (RRR = 1.29, 95% CI 1.11–1.51) trajectory groups.

**Fig. 3:**
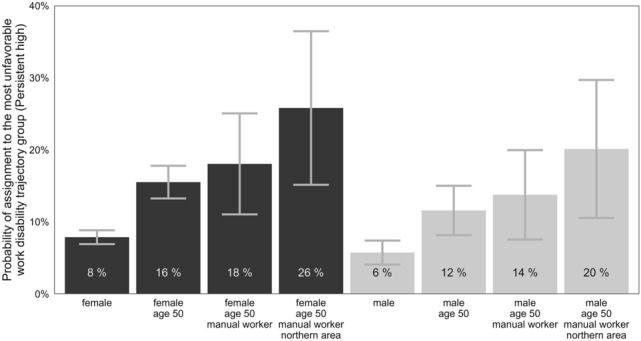
Estimated marginal probabilities from the multinomial regression model showing the combined effects of sociodemographic characteristics on persistent high-trajectory group membership

To measure the combined effects of sociodemographic characteristics on trajectory group membership, we calculated marginal probabilities of assignment to the most unfavorable, persistent high group from the results of the regression analysis. More specifically, we estimated the probability of assignment to the persistent high work disability trajectory group among males and females by age (30 or 50 years), occupational status (upper-level employees or manual workers), and geographical area. The presence of multiple risk factors substantially increased the probability of assignment to the persistent high trajectory group (see Fig. 3). For example, for a 50-year-old female manual worker living in Northern Finland, the probability of being assigned to the persistent high group was 25.8% (95% CI 15.2–36.5), whereas, for a 30-year-old upper-level male employee living in Southern Finland, the probability was 1.4% (95% CI 0.9–2.1). The full details of all possible combinations are shown in Supplementary Table 1.

## Discussion

We investigated sociodemographic and regional factors that were associated with depression or anxiety disorder-related work disability trajectories in a nationally representative sample of working-age Finnish receiving psychotherapy. Four different trajectories were found: most of the cohort (72%) showed stable very low work disability during the follow-up and one-tenth of the cohort (11%) showed a decrease in work disability. Of the sample, 7% showed persistent high and 9% showed persistent low work disability over  a year before and 4 years after the onset of psychotherapy. Older age, female gender, lower occupational status and living in more sparsely populated areas such as the Eastern area of Finland were associated with higher and persistent work disability. Individuals with a persistent high and persistent low work disability received rehabilitative psychotherapy slightly longer than individuals in other groups.

In this register study, those whose work disability remained very low can be considered a favorable group suggesting that therapy maintains these clients’ ability to work as intended in the legislation of public rehabilitation services. The results are in line with previous studies suggesting that younger age, higher occupational status and shorter length of preceding disability were associated with better outcomes in psychotherapy [37, 38] and vocational rehabilitation [39] and with earlier return to work after mental disorder -related sick leave [40, 41]. Similarly, those whose work disability decreased during the therapy, labeled as the decrease group, can also be considered as a favorable group as workability outcomes improved in association with rehabilitative psychotherapy. On the contrary, in two other groups, work disability remained poor or very poor in association with psychotherapy. Female gender and living in sparsely populated areas of Finland stand out as risk factors for prolonged work disability. Notably, work disability in the least favorable persistent high group was higher at the onset of therapy than in the other groups and multiple risk factors were associated with the increased risk of persistent high work disability.

According to our hypothesis, low occupational status was associated with persistent work disability, the results point out work-related contextual differences in the ability to benefit from person-centered rehabilitative psychotherapy. There is cumulative evidence that the burden of adverse work characteristics on work disability and early exit from working life is pronounced among low occupational status individuals [18, 19, 40, 42, 43]. Thus, as the impact of working life structures on work disability has shown to differ among occupational classes, persistent work disability trajectories seen in our study may reflect occupation-related possibilities for rehabilitation rather than individuals’ workability per se. i.e., possibilities for job accommodations that support working despite the mental health condition may be better in upper occupational positions. The association between occupational status and work disability may be also enhanced by gendered occupational statuses: it has been shown that among women low occupational status is associated with increased mental ill health [44]. Thus, the influence of adverse work-related risk factors on ability to benefit from psychotherapy may be more pronounced among women as our results suggest. Furthermore, there is some evidence that a combination of various practical, psychological, and cultural barriers influences treatment engagement and gaining from psychotherapy among employees with lower occupational status [45, 46]. In our study, those with persistent high work disability had a slightly longer treatment duration when compared with the stable very low group. This might reflect the practical challenges in attending regular treatment sessions among manual workers and thus lengthening the treatment. Furthermore, in their recent study, Williams et al. also showed that those in routine or manual work found their work less meaningful than those in higher occupational classes [47]. Thus, these characteristics related to labor market structures, possibilities to engage in rehabilitation and factors influencing working-life attachment may jointly increase the risk of prolonged disability due to mental health reasons among employees with lower status. Thus, the stagnation in trajectories of these groups suggests that individual-based rehabilitation is not sufficient to support workability in cases with persistent disability. Reducing prolonged disability due to mental disorders might require broader interventions that combine individual-based rehabilitation and occupational adjustments.

Although two recent studies have suggested that rehabilitative psychotherapy associates with better labor market outcomes [31, 32], there has been only scarce information on factors influencing individuals’ outcomes in association with long-term psychotherapy comparable to our study. Results from Helsinki Psychotherapy Study suggest that similarly to our study, most clients’ work disabilities remain stable or improved during long-term psychotherapy [15]. In a 10-year follow-up, workability among clients receiving long-term psychotherapy improved significantly more than in groups with shorter (12–20 sessions during 5–8 months) psychotherapy [15]. However, after 10 years of follow-up, only about 60% were in remission and 33% of them used some auxiliary psychiatric treatment during the follow-up. These results made the authors suggest, that the treatment given during that long period was not particularly effective in providing long-term benefits for individuals with relatively long-standing work disability related to depression or anxiety disorders [15]. Similar conclusions can be drawn from our results where high baseline disability was associated with poor outcomes during follow-up.

Thus, a persistent high work disability should be considered in a broader context of health and correlates that modify health outcomes. Mental disorders have been associated with physical comorbidity [48, 49] and the number of comorbidities was associated with work disability in terms of low work productivity, frequent sick leaves and a lower rate of return to work after sick leave [40, 50]. These findings were especially prominent in older age groups and related to low socioeconomic status [12, 40, 51–53]. Furthermore, the incidence rates of depressive and anxiety disorders have shown to be approximately two-fold among women in comparison to men [54, 55], in line with the overall greater burden of ill health and functional problems among women [56]. As previously outlined, together with the severity of the condition, psychosocial work characteristics have been indicated to predict sick leave outcomes [19]. Thus, the relevance of the treatment for those with persistent high work disability should be evaluated more carefully as poor baseline functioning, comorbidities and workplace attitudes towards disorder might hinder an individual’s possibilities to benefit from mere psychotherapy [23, 24, 57] and correlates of occupational position might influence this association.

As a novel finding, our study shows that also the work disability associated with psychotherapeutic treatment differs between geographical areas. It might be that the lower regional availability of psychotherapy in sparsely populated Northern and Eastern areas of Finland [58] might hinder receiving psychotherapy timely in these areas. Furthermore, when considering the regional differences in the impact of common mental disorders on losses of workability, quality of life, increased health expenditures and mortality, Northern and Eastern areas show the largest burden of health [59]. This might reflect larger regional differences in mental health services that might result in too-late rehabilitation for individuals in persistent high disability groups. Such assumptions are supported by a recent study by Karolaakso et al. who found corresponding evidence on regional differences in risk of disability pension related to mood disorders. Although one could suggest that the influence of work-related risk factors on work disability is more pronounced in more disadvantaged areas with fewer occupational opportunities, their results were not fully explained by socioeconomic factors [21]. Thus, together these results seem to illustrate the divergent functioning of mental health care services across different geographical areas.

This study has several strengths. It is based on reliable register-based national data combined from multiple sources and with a considerably long follow-up period. We combined information on individuals’ work disability months based on medical diagnoses, information on psychotherapy use and information on sociodemographic factors recorded independently from each other. A population-based cohort of all psychotherapy users during the research period gave us satisfactory power for the analyses and allowed us to assess the latent sub-groups with similar work disability trajectories over time [60] in a representative sample of Finnish employees. Further studies should widen the scope of the target group also to students or self-employed persons to assess diversity in response to treatment in such groups of psychotherapy clients.

Our study also has limitations. Given the descriptive nature of this study without comparison groups (those who did not receive any treatment or received some other type of treatment), the trajectories found in this study may not fully reflect the response to the treatment but also, as was discussed above, result from regional differences in the labor market structures and the organization of health care. Although the Social Insurance Institution register covers most sickness absence days due to mental disorders in Finland, our work disability outcome was somewhat crude as information on shorter than ten-day sick leaves was not available. Also, rehabilitative psychotherapy is targeted at those who already are disabled to work but also at those at risk for work disability. Thus, the results may not reflect the whole spectrum of work disability or the association between the treatment and work disability risk factors. Furthermore, the follow-up related to the treatment was relatively short and we were unable to assess the stability of the results. We looked only at the association between psychotherapy use and work disability trajectories and thus we cannot make further assumptions about the effectiveness of various types of treatment on work disability. Although we were unable to assess the influence of more detailed characteristics of the treatment and individuals on the outcome, differencing work disability trajectories raise the question of whether the type of treatment or treatment intensity reflected the patients’ needs. Also, approximately 90% of the rehabilitation psychotherapies have been shown to be related to depressive and anxiety disorders [61] suggesting a high correlation between reasons for work disability and psychotherapy. However, we cannot rule out that some individuals included in our study may have received psychotherapy for some other reason (for example due to eating disorders). Further studies are thus needed to assess the coverage and needs of various target groups and the choice of psychotherapy. In addition, suitability to the treatment should be considered based on personalized needs including both personal [62, 63] and contextual factors such as work-related characteristics to understand their interaction on workability outcomes.

## Conclusions

This population-level register-based study shows that several sociodemographic characteristics are associated with subsequent work disability trajectories among individuals who attend long-term psychotherapy. Older age, female gender, lower occupational status, and living in sparsely populated geographical areas of Finland were associated with cumulatively greater risk for persistent high work disability. These findings indicate that despite the equal admission criteria for the treatment there are inequalities in achieving improvement in workability in association with long-term rehabilitative psychotherapy. Further studies should focus on these factors reflecting societal barriers to recovery of workability in conjunction with morbidity and work-related characteristics.

### Supplementary Information

Below is the link to the electronic supplementary material.

Supplementary file1 (DOCX 510 KB)

## Data Availability

Data in this study were used under its’ license approved by Statistics Finland, Social Security Institution of Finland, Finnish Centre for Pensions. They are not publicly available. Currently, the Finnish Social and Health Data Permit Authority (Findata) coordinates the permissions for similar datasets, and they are available to researchers under permission (https://findata.fi/en/). The THL’S morbidity indexes are publicly available from the Sotkanet Indicator Bank, an information portal provided by THL (https://sotkanet.fi/sotkanet/en/haku?g=284). Provenance and peer review: not commissioned; externally peer-reviewed.
