# Supplementary material for: The association of sociodemographic characteristics with work disability trajectories during and following long-term psychotherapy: a longitudinal register study
Source: Soc Psychiatry Psychiatr Epidemiol. 2023 Jul 11;59(4):621–30. doi: 10.1007/s00127-023-02523-y (PMC10960900; doi:10.1007/s00127-023-02523-y)

Supplementary material for the original paper entitled The association of sociodemographic characteristics with work disability trajectories during and following long-term psychotherapy: a longitudinal register study by Sanna Selinheimo1, Kia Gluschkoff 1, 2, Johanna Kausto1, Jarno Turunen1, Aki Koskinen1 and Ari Väänänen1

1 Finnish Institute of Occupational Health, Helsinki, Finland

2 Department of Psychology and Logopedics, University of Helsinki

*Corresponding author: Sanna Selinheimo, [sanna.selinheimo@ttl.fi](mailto:sanna.selinheimo@ttl.fi)

**Supplementary Table 1.** Estimated marginal probability of assignment to the most unfavorable work disability trajectory group (Persistent high) among males and females by age (30 or 50 years), occupational status (high or low), and geographical area.

| Gender | Occupational status | | Age 30 years |  | Age 50 years |  |
| --- | --- | --- | --- | --- | --- | --- |
|  |  | | Probability | 95% CI | Probability | 95% CI |
| Male | Upper-level employee |  |  |  |  |  |
|  |  | Southern | 1.4 % | 0.9 to 2.1 | 7.2 % | 4.6 to 10.0 |
|  |  | Western | 2.3 % | 1.1 to 3.6 | 11.0 % | 6.3 to 15.8 |
|  |  | Central | 2.6 % | 1.3 to 3.8 | 11.6 % | 6.9 to 16.3 |
|  |  | Eastern | 3.4 % | 1.8 to 4.9 | 15.0 % | 9.5 to 20.6 |
|  |  | Northern | 3.5 % | 1.8 to 5.2 | 15.4 % | 9.4 to 21.4 |
|  | Manual worker |  |  |  |  |  |
|  |  | Southern | 2.2 % | 0.9 to 3.4 | 10.0 % | 4.8 to 15.1 |
|  |  | Western | 3.4 % | 1.4 to 5.5 | 14.9 % | 7.2 to 22.5 |
|  |  | Central | 3.6 % | 1.6 to 5.7 | 15.2 % | 7.6 to 22.8 |
|  |  | Eastern | 4.8 % | 2.0 to 7.5 | 19.5 % | 10.2 to 28.8 |
|  |  | Northern | 4.9 % | 2.1 to 7.8 | 20.1 % | 10.5 to 29.7 |
|  |  |  |  |  |  |  |
| Female | Upper-level employee |  |  |  |  |  |
|  |  | Southern | 2.2 % | 1.4 to 2.9 | 10.2 % | 7.5 to 12.9 |
|  |  | Western | 3.5 % | 2.0 to 5.0 | 15.2 % | 10.2 to 20.2 |
|  |  | Central | 3.7 % | 2.2 to 5.2 | 15.6 % | 10.7 to 20.5 |
|  |  | Eastern | 4.9 % | 3.0 to 6.7 | 20.0 % | 14.5 to 25.5 |
|  |  | Northern | 5.1 % | 3.0 to 7.1 | 20.6 % | 14.5 to 26.7 |
|  | Manual worker |  |  |  |  |  |
|  |  | Southern | 3.1 % | 1.5 to 4.7 | 13.5 % | 7.4 to 19.6 |
|  |  | Western | 4.9 % | 2.3 to 7.6 | 19.7 % | 11.7 to 28.7 |
|  |  | Central | 5.1 % | 2.4 to 7.8 | 19.6 % | 10.9 to 28.2 |
|  |  | Eastern | 6.7 % | 3.2 to 10.1 | 24.8 % | 14.6 to 35.1 |
|  |  | Northern | 7.0 % | 3.3 to 10.7 | 25.8 % | 15.2 to 36.5 |

Southern = Helsinki University Hospital district; Western = Turku University Hospital district; Central = Tampere University Hospital district; Eastern = Kuopio University Hospital district; Northern = Oulu University Hospital district

**The model selection process in group-based trajectory modeling**

traj, var(dmonths0 dmonths1 dmonths2 dmonths3 dmonths4) indep(t_0 t_1 t_2 t_3 t_4) model(zip) order(2 2) iorder(0)

Standard T for H0:

Group Parameter Estimate Error Parameter=0 Prob > |T|

1 Intercept -1.15015 0.09187 -12.520 0.0000

Linear -0.13961 0.11057 -1.263 0.2068

Quadratic -0.08918 0.03204 -2.784 0.0054

2 Intercept 1.43122 0.02433 58.819 0.0000

Linear 0.21766 0.02771 7.854 0.0000

Quadratic -0.03853 0.00657 -5.860 0.0000

Alpha0 0.46850 0.03780 12.393 0.0000

Group membership

1 (%) 72.47888 0.89532 80.953 0.0000

2 (%) 27.52112 0.89532 30.739 0.0000

BIC=-13128.69 (N=18025) BIC=-13122.25 (N=3605) AIC=-13097.49 ll= -13089.49

Entropy = 0.755


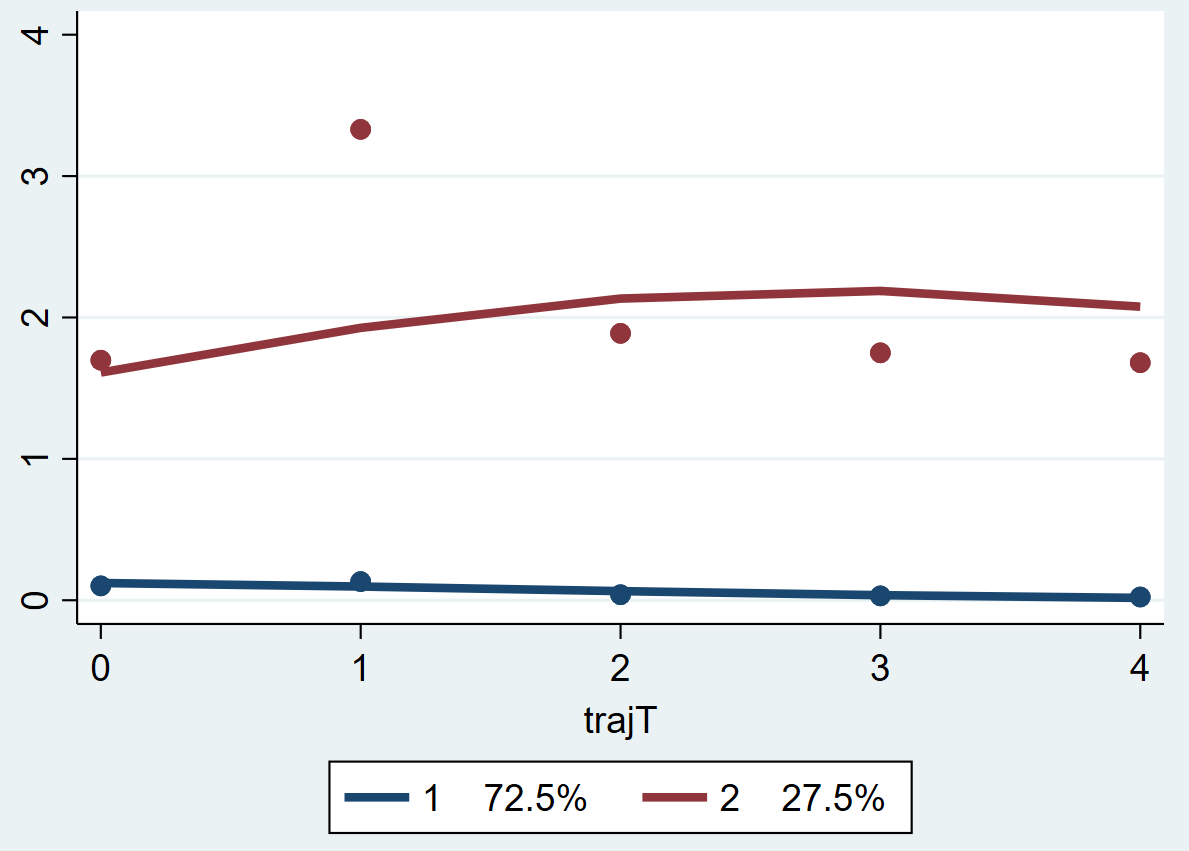


traj, var(dmonths0 dmonths1 dmonths2 dmonths3 dmonths4) indep(t_0 t_1 t_2 t_3 t_4) model(zip) order(2 2 2) iorder(0)

Standard T for H0:

Group Parameter Estimate Error Parameter=0 Prob > |T|

1 Intercept -2.31811 0.15096 -15.356 0.0000

Linear 1.80058 0.45339 3.971 0.0001

Quadratic -1.43387 0.33650 -4.261 0.0000

2 Intercept 0.91018 0.05526 16.472 0.0000

Linear 0.20346 0.08538 2.383 0.0172

Quadratic -0.16712 0.02296 -7.278 0.0000

3 Intercept 1.61631 0.03007 53.744 0.0000

Linear 0.22312 0.03302 6.758 0.0000

Quadratic -0.04068 0.00761 -5.343 0.0000

Alpha0 0.28687 0.03800 7.549 0.0000

Group membership

1 (%) 62.30823 1.13437 54.928 0.0000

2 (%) 22.77108 1.02544 22.206 0.0000

3 (%) 14.92069 0.77244 19.316 0.0000

BIC=-12669.40 (N=18025) BIC=-12659.74 (N=3605) AIC=-12622.60 ll= -12610.60

Entropy = 0.704


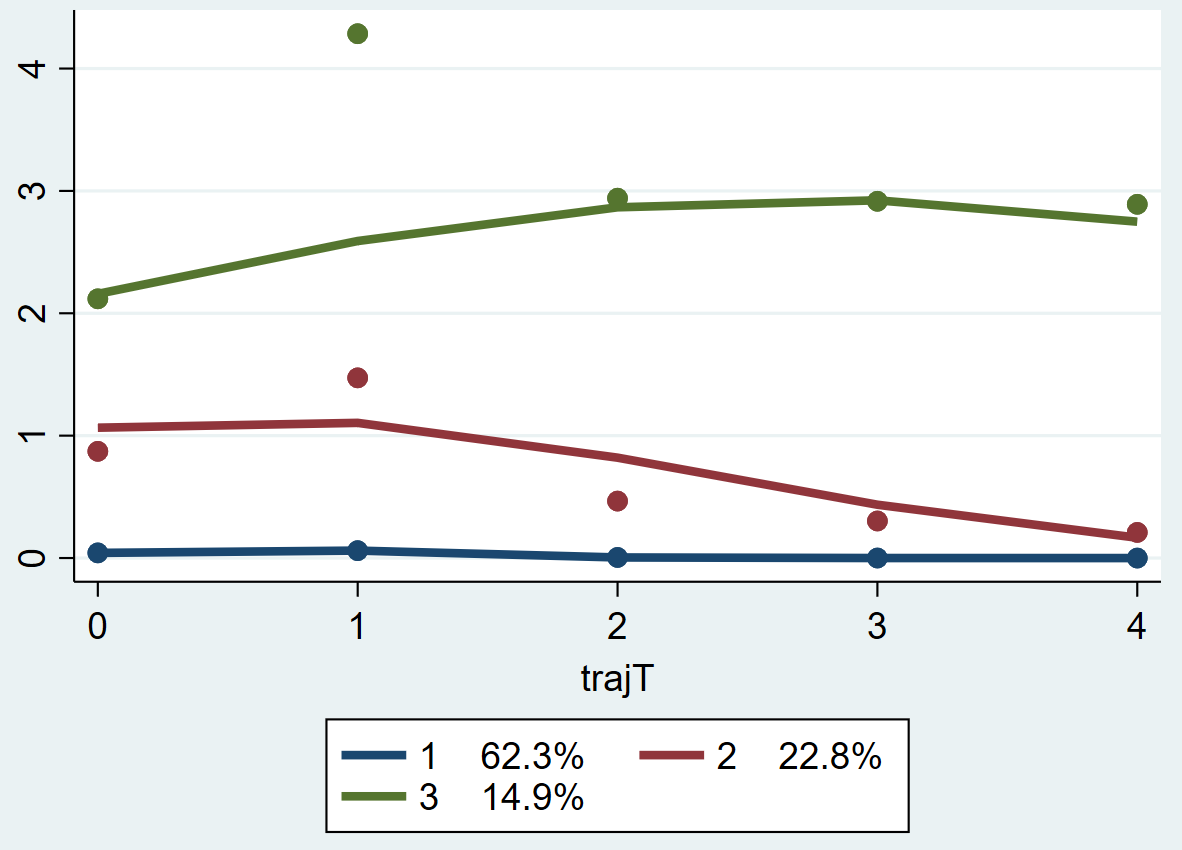


traj, var(dmonths0 dmonths1 dmonths2 dmonths3 dmonths4) indep(t_0 t_1 t_2 t_3 t_4) model(zip) order(2 2 2 2) iorder(0)

Standard T for H0:

Group Parameter Estimate Error Parameter=0 Prob > |T|

1 Intercept 0.57001 0.10552 5.402 0.0000

Linear 0.42213 0.11734 3.597 0.0003

Quadratic -0.12075 0.02837 -4.256 0.0000

2 Intercept -2.68293 0.16443 -16.317 0.0000

Linear -0.09506 0.19758 -0.481 0.6304

Quadratic -0.05308 0.05281 -1.005 0.3149

3 Intercept 0.96399 0.09309 10.355 0.0000

Linear 2.46838 0.21860 11.292 0.0000

Quadratic -2.10855 0.17873 -11.797 0.0000

4 Intercept 1.69446 0.03407 49.733 0.0000

Linear 0.17266 0.03645 4.737 0.0000

Quadratic -0.02863 0.00828 -3.458 0.0005

Alpha0 -0.07764 0.04336 -1.791 0.0734

Group membership

1 (%) 9.91427 0.68902 14.389 0.0000

2 (%) 62.65317 1.14770 54.590 0.0000

3 (%) 16.92946 0.96446 17.553 0.0000

4 (%) 10.50310 0.66622 15.765 0.0000

BIC=-12461.13 (N=18025) BIC=-12448.25 (N=3605) AIC=-12398.73 ll= -12382.73

Entropy = 0.721


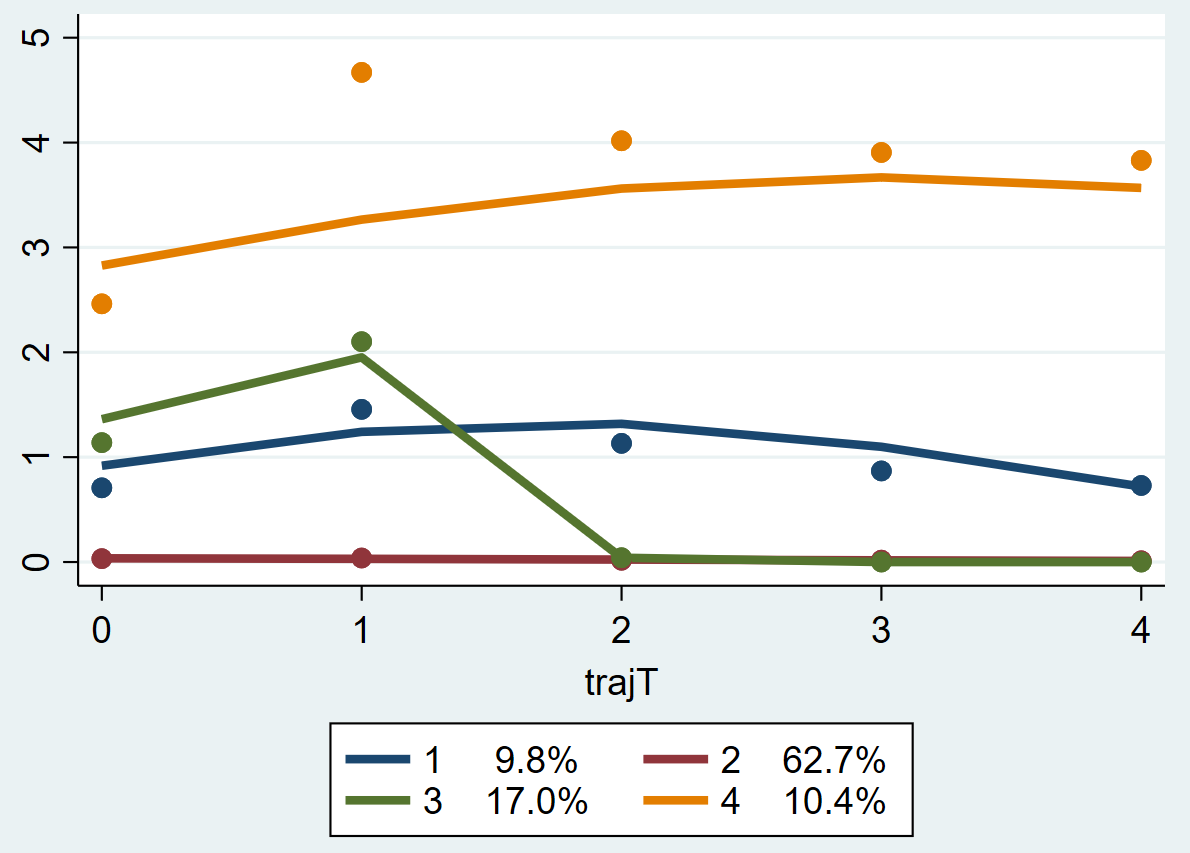


traj, var(dmonths0 dmonths1 dmonths2 dmonths3 dmonths4) indep(t_0 t_1 t_2 t_3 t_4) model(zip) order(2 2 2 2 2) iorder(0)

Standard T for H0:

Group Parameter Estimate Error Parameter=0 Prob > |T|

1 Intercept -0.94171 0.26500 -3.554 0.0004

Linear 0.19196 0.26888 0.714 0.4753

Quadratic -0.07810 0.05305 -1.472 0.1410

2 Intercept -3.83255 0.94425 -4.059 0.0000

Linear 3.95090 3.77628 1.046 0.2955

Quadratic -3.38207 3.61985 -0.934 0.3502

3 Intercept 0.77343 0.11147 6.939 0.0000

Linear 0.36386 0.11343 3.208 0.0013

Quadratic -0.08850 0.02941 -3.009 0.0026

4 Intercept 0.99747 0.10443 9.551 0.0000

Linear 2.53447 0.23617 10.732 0.0000

Quadratic -2.16614 0.20021 -10.819 0.0000

5 Intercept 1.71947 0.03715 46.281 0.0000

Linear 0.17878 0.03832 4.666 0.0000

Quadratic -0.02992 0.00871 -3.435 0.0006

Alpha0 -0.01028 0.04399 -0.234 0.8152

Group membership

1 (%) 14.59638 3.10264 4.705 0.0000

2 (%) 51.25284 3.18165 16.109 0.0000

3 (%) 7.82088 0.71811 10.891 0.0000

4 (%) 16.75118 1.19224 14.050 0.0000

5 (%) 9.57871 0.78237 12.243 0.0000

BIC=-12440.91 (N=18025) BIC=-12424.82 (N=3605) AIC=-12362.92 ll= -12342.92

Entropy = 0.597


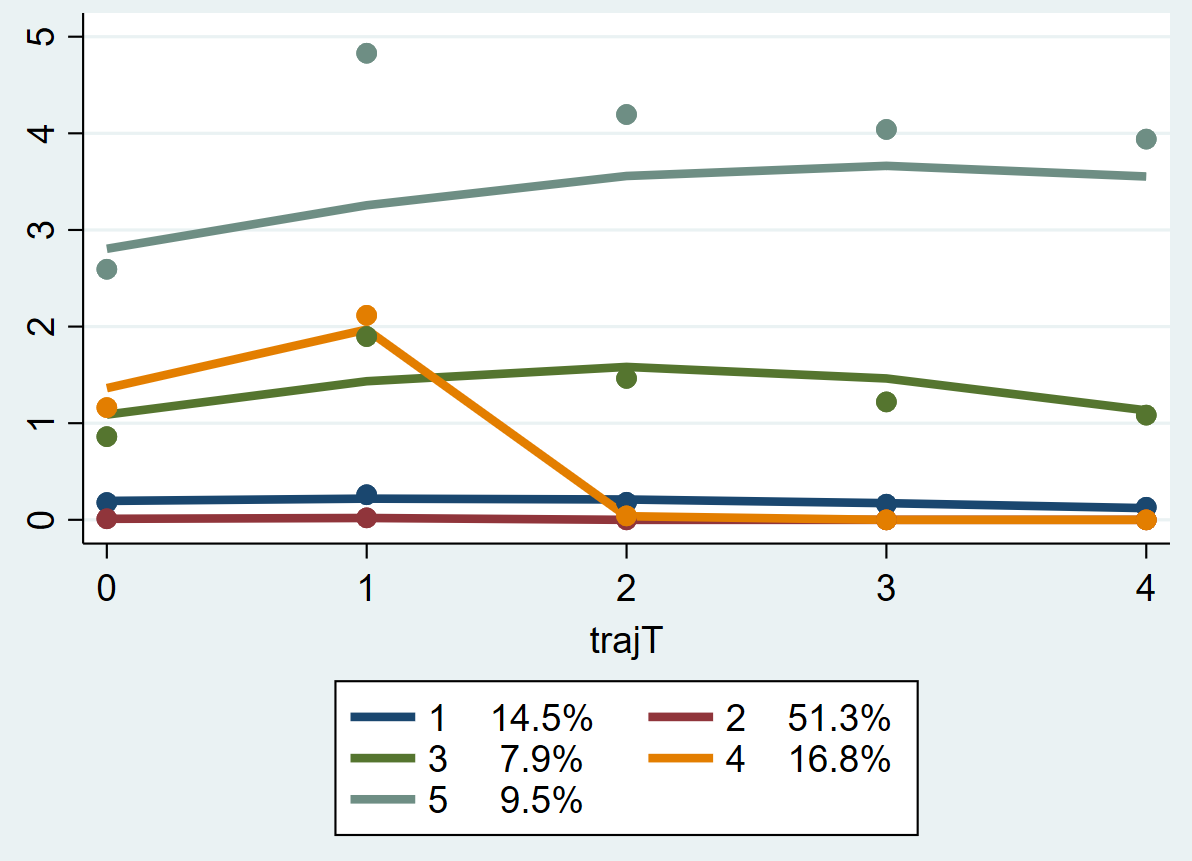


Based on the results above, a 4-group solution was selected because the 5-group solution does not capture any new distinct features (as can be seen from the figure on the previous page, groups 1 and 2 are very similar). Next, the solution was refined for a better fit.

traj, var(dmonths0 dmonths1 dmonths2 dmonths3 dmonths4) indep(t_0 t_1 t_2 t_3 t_4) model(zip) order(0 2 2 2) iorder(0)

Standard T for H0:

Group Parameter Estimate Error Parameter=0 Prob > |T|

1 Intercept 0.45013 0.06452 6.977 0.0000

2 Intercept -2.97733 0.23735 -12.544 0.0000

Linear 8.09985 128.55473 0.063 0.9498

Quadratic -7.51654 128.55533 -0.058 0.9534

3 Intercept 0.87705 0.10387 8.444 0.0000

Linear 2.20906 0.19275 11.461 0.0000

Quadratic -1.71270 0.13009 -13.165 0.0000

4 Intercept 1.68367 0.03206 52.519 0.0000

Linear 0.15715 0.03427 4.586 0.0000

Quadratic -0.02527 0.00791 -3.197 0.0014

Alpha0 0.04147 0.04332 0.957 0.3384

Group membership

1 (%) 12.29523 0.75034 16.386 0.0000

2 (%) 58.69806 1.18866 49.382 0.0000

3 (%) 17.41334 1.04706 16.631 0.0000

4 (%) 11.59337 0.67330 17.219 0.0000

BIC=-12513.00 (N=18025) BIC=-12501.74 (N=3605) AIC=-12458.41 ll= -12444.41

Warning: Variance matrix is nonsymmetric or highly singular.

Entropy = 0.678

This model below has significant cubic polynomials in three groups, but according to the visual inspection, the fit is not good.

traj, var(dmonths0 dmonths1 dmonths2 dmonths3 dmonths4) indep(t_0 t_1 t_2 t_3 t_4) model(zip) order(0 3 3 3) iorder(0)

Standard T for H0:

Group Parameter Estimate Error Parameter=0 Prob > |T|

1 Intercept -3.14345 0.10583 -29.704 0.0000

2 Intercept 0.92481 0.08174 11.314 0.0000

Linear 3.37438 0.47617 7.087 0.0000

Quadratic -3.73537 0.55316 -6.753 0.0000

Cubic 0.65632 0.10548 6.222 0.0000

3 Intercept 0.28250 0.15213 1.857 0.0633

Linear 1.50945 0.27450 5.499 0.0000

Quadratic -0.76751 0.15469 -4.962 0.0000

Cubic 0.10751 0.02466 4.359 0.0000

4 Intercept 1.67913 0.04138 40.579 0.0000

Linear 0.36923 0.08516 4.335 0.0000

Quadratic -0.15286 0.05064 -3.018 0.0025

Cubic 0.01998 0.00813 2.457 0.0140

Alpha0 -0.08191 0.04491 -1.824 0.0682

Group membership

1 (%) 61.90751 1.20547 51.355 0.0000

2 (%) 19.34441 1.09420 17.679 0.0000

3 (%) 9.28338 0.71983 12.897 0.0000

4 (%) 9.46470 0.85467 11.074 0.0000

BIC=-12453.43 (N=18025) BIC=-12439.75 (N=3605) AIC=-12387.14 ll= -12370.14

Entropy = 0.718


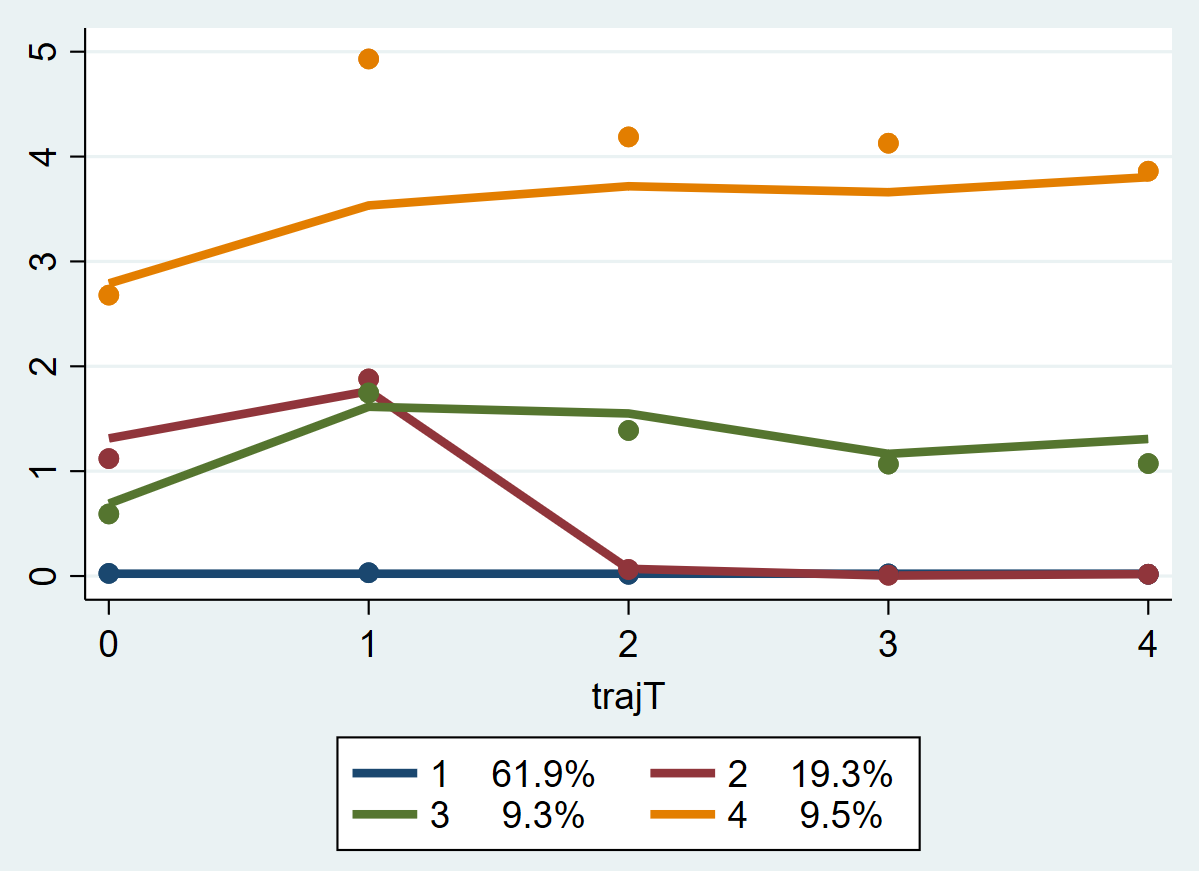


The iorder option was changed to 3: traj, var(dmonths0 dmonths1 dmonths2 dmonths3 dmonths4) indep(t_0 t_1 t_2 t_3 t_4) model(zip) order(0 3 3 3) iorder(3)

Standard T for H0:

Group Parameter Estimate Error Parameter=0 Prob > |T|

1 Intercept -3.02831 0.09544 -31.730 0.0000

2 Intercept 1.04147 0.07844 13.277 0.0000

Linear 3.79115 0.40327 9.401 0.0000

Quadratic -4.42138 0.47121 -9.383 0.0000

Cubic 0.80322 0.09369 8.573 0.0000

3 Intercept 0.53256 0.12175 4.374 0.0000

Linear 1.01684 0.25502 3.987 0.0001

Quadratic -0.55428 0.15439 -3.590 0.0003

Cubic 0.08284 0.02490 3.326 0.0009

4 Intercept 1.65992 0.04487 36.994 0.0000

Linear 0.41461 0.08969 4.623 0.0000

Quadratic -0.17630 0.05242 -3.363 0.0008

Cubic 0.02335 0.00837 2.789 0.0053

Alpha0 0.20478 0.06830 2.998 0.0027

Alpha1 -1.19314 0.16780 -7.111 0.0000

Alpha2 0.63514 0.11546 5.501 0.0000

Alpha3 -0.08493 0.01984 -4.279 0.0000

Group membership

1 (%) 63.18930 1.15805 54.565 0.0000

2 (%) 17.99478 1.04540 17.213 0.0000

3 (%) 9.44418 0.72051 13.108 0.0000

4 (%) 9.37174 0.78276 11.973 0.0000

BIC=-12432.29 (N=18025) BIC=-12416.19 (N=3605) AIC=-12354.29 ll= -12334.29

Entropy = 0.731


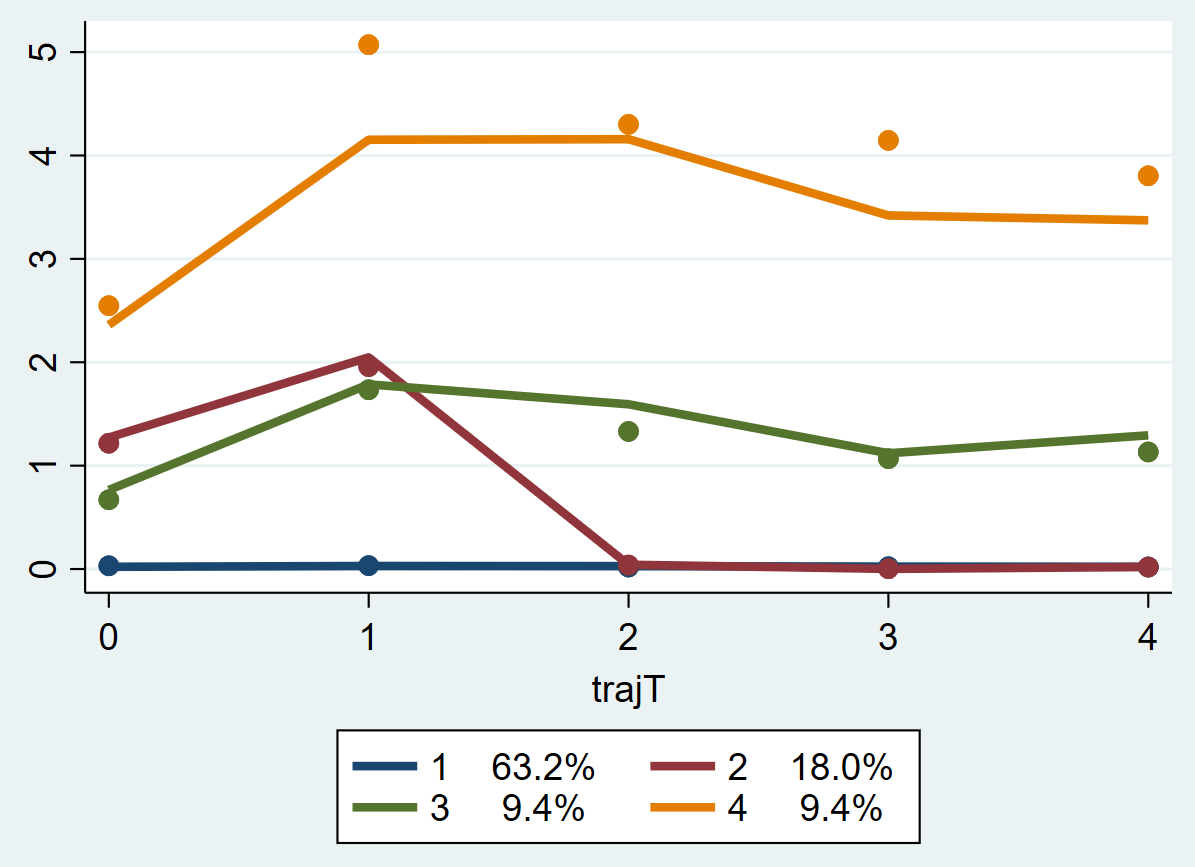


Different model specifications were tested (many of which did not converge), and the model below had a good fit. However, in group 2, the polynomials had become nonsignificant.

traj, var(dmonths0 dmonths1 dmonths2 dmonths3 dmonths4) indep(t_0 t_1 t_2 t_3 t_4) model(zip) order(0 3 3 3) iorder(0 0 0 3)

Standard T for H0:

Group Parameter Estimate Error Parameter=0 Prob > |T|

1 Intercept 0.86285 0.05962 14.474 0.0000

2 Intercept -0.33581 0.15978 -2.102 0.0356

Linear 5.04261 1.643e+005 0.000 1.0000

Quadratic 2.22919 1.093e+005 0.000 1.0000

Cubic -6.47935 2.029e+005 -0.000 1.0000

3 Intercept 1.50114 0.05761 26.059 0.0000

Linear 1.88623 0.21758 8.669 0.0000

Quadratic -1.99385 0.24068 -8.284 0.0000

Cubic 0.33365 0.04744 7.033 0.0000

4 Intercept 1.51681 0.04533 33.460 0.0000

Linear 0.56865 0.08855 6.422 0.0000

Quadratic -0.22815 0.05053 -4.515 0.0000

Cubic 0.02902 0.00799 3.631 0.0003

1 Alpha0 0.63838 0.09081 7.030 0.0000

2 Alpha0 1.93109 0.08088 23.877 0.0000

3 Alpha0 0.67072 0.15704 4.271 0.0000

4 Alpha0 -0.10224 0.14465 -0.707 0.4797

Alpha1 -2.47146 0.42055 -5.877 0.0000

Alpha2 1.10747 0.27062 4.092 0.0000

Alpha3 -0.13230 0.04394 -3.011 0.0026

Group membership

1 (%) 13.61640 1.32568 10.271 0.0000

2 (%) 63.58447 1.47460 43.120 0.0000

3 (%) 15.78669 1.81271 8.709 0.0000

4 (%) 7.01244 0.55175 12.709 0.0000

BIC=-12312.96 (N=18025) BIC=-12294.45 (N=3605) AIC=-12223.26 ll= -12200.26

Entropy = 0.660


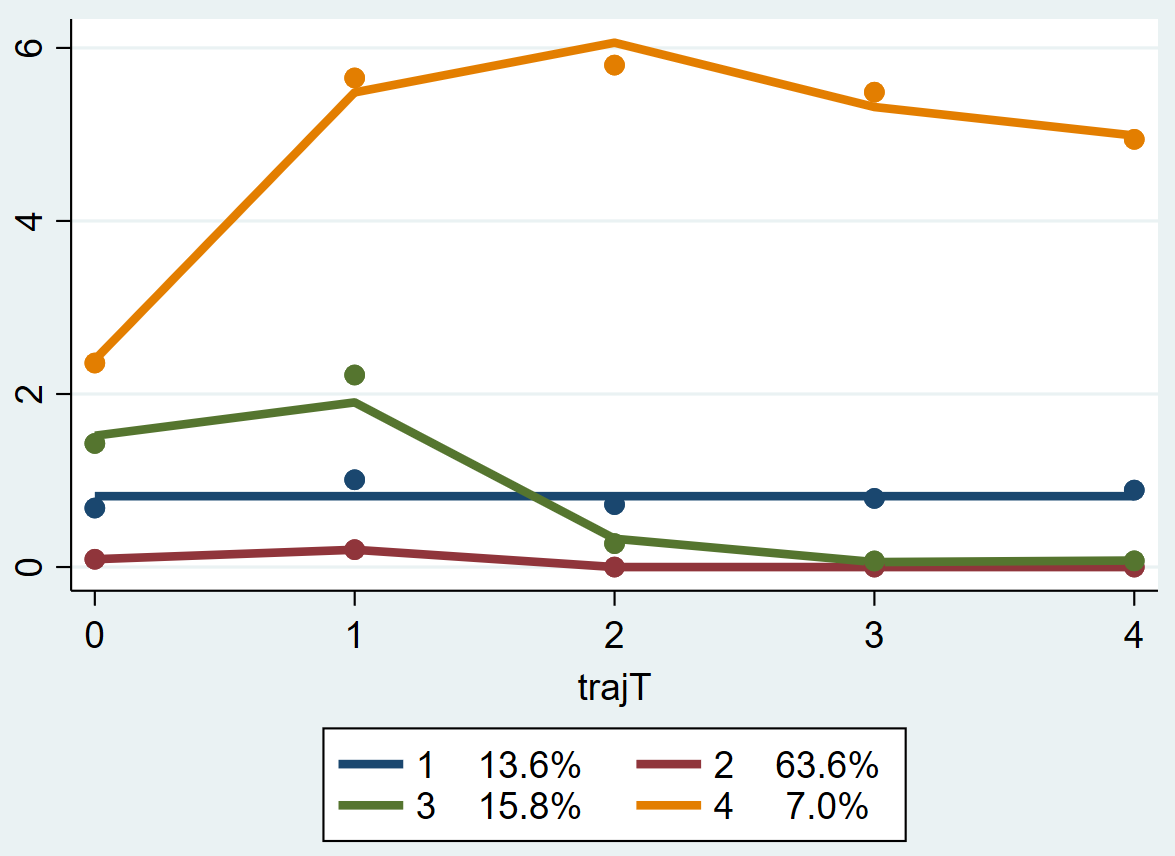


When the nonsignificant polynomials were removed, the best fit was achieved. The average posterior probabilities in this final model were 0.85 (group 1), 0.88 (group 2) , 0.75 (group 3) and 0.94 (group 4).

traj, var(dmonths0 dmonths1 dmonths2 dmonths3 dmonths4) indep(t_0 t_1 t_2 t_3 t_4) model(zip) order(0 2 3 3) iorder(0 0 0 3)

Standard T for H0:

Group Parameter Estimate Error Parameter=0 Prob > |T|

1 Intercept 0.77118 0.05470 14.097 0.0000

2 Intercept -0.41031 0.17419 -2.356 0.0185

Linear 3.21644 0.51846 6.204 0.0000

Quadratic -2.32190 0.44884 -5.173 0.0000

3 Intercept 1.49152 0.05508 27.079 0.0000

Linear 2.58358 0.41648 6.203 0.0000

Quadratic -2.84457 0.49187 -5.783 0.0000

Cubic 0.49339 0.09571 5.155 0.0000

4 Intercept 1.52029 0.04450 34.163 0.0000

Linear 0.54366 0.08755 6.210 0.0000

Quadratic -0.22130 0.04958 -4.464 0.0000

Cubic 0.02867 0.00783 3.663 0.0002

1 Alpha0 0.79425 0.07770 10.222 0.0000

2 Alpha0 2.02210 0.08421 24.011 0.0000

3 Alpha0 -0.02434 0.23902 -0.102 0.9189

4 Alpha0 -0.02790 0.13727 -0.203 0.8389

Alpha1 -2.65836 0.40651 -6.540 0.0000

Alpha2 1.22742 0.26003 4.720 0.0000

Alpha3 -0.14994 0.04201 -3.570 0.0004

Group membership

1 (%) 16.66903 1.39695 11.932 0.0000

2 (%) 64.91154 1.42746 45.473 0.0000

3 (%) 10.90767 1.60512 6.796 0.0000

4 (%) 7.51177 0.55725 13.480 0.0000

BIC=-12314.00 (N=18025) BIC=-12296.29 (N=3605) AIC=-12228.20 ll= -12206.20

Entropy = 0.697


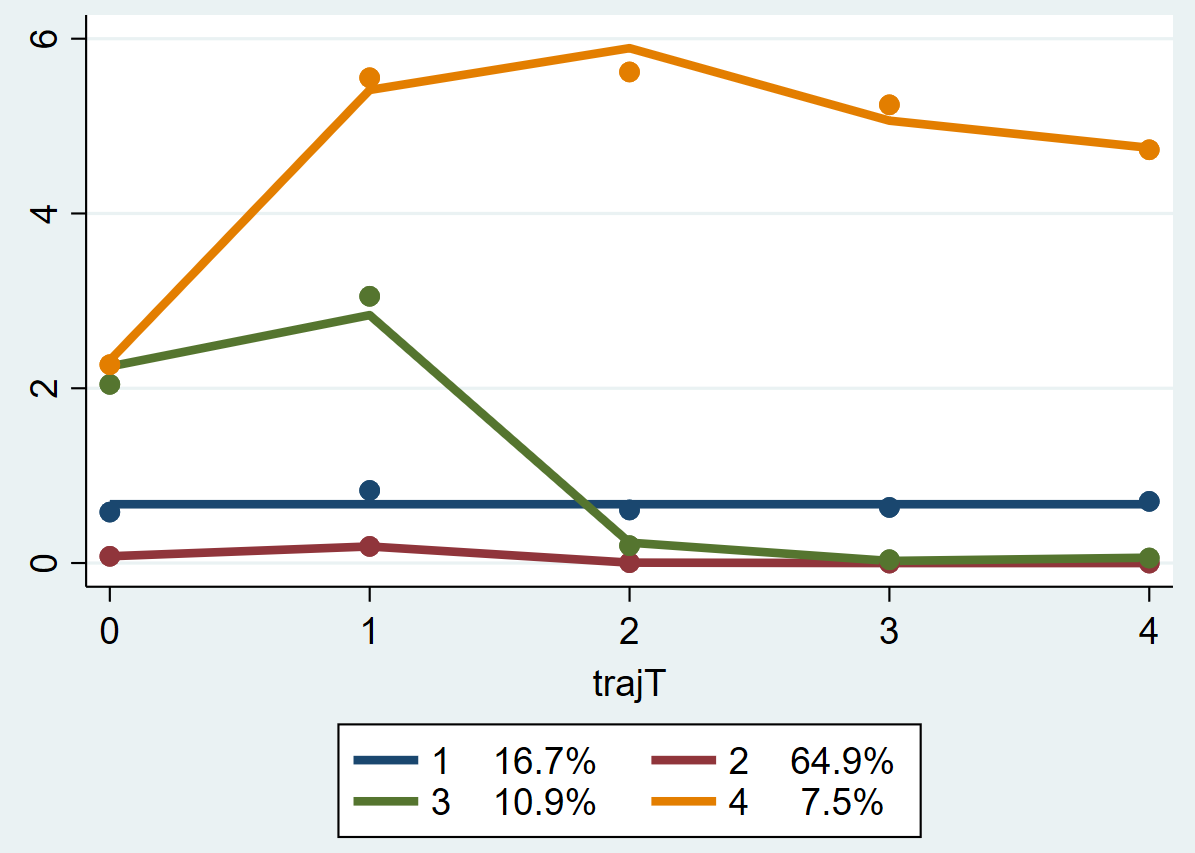

Supplement: Supplementary file 1 — Supplementary file1 (DOCX 510 KB) [file 127_2023_2523_MOESM1_ESM.docx]
